# Reversible Pisa syndrome associated to subdural haematoma: case-report

**DOI:** 10.1186/1471-2377-14-149

**Published:** 2014-08-14

**Authors:** Pasquale Marchione, Aldo Spallone, Marcella Valente, Cristiano Giannone, Floriana De Angelis, Giuseppe Meco

**Affiliations:** 1 Department of Clinical Neurosciences, Neurological Center of Latium – Institute of Neurosciences, Via Patrica 15, 00178 Rome, Italy; 2 Department of Medical and Surgical Sciences and Biotechnologies – Section of Neurology, Sapienza, University of Rome, Viale dell’Università 30, 00185 Rome, Italy; 3 Parkinson’s Centre and Research Centre of Social Diseases (CIMS), Department of Neurology and Psychiatry, Sapienza University of Rome, Viale dell’Università 30, 00185 Rome, Italy

**Keywords:** Pisa Syndrome, Subdural Haematoma, Parkinsonism, Cholinergic-dopaminergic imbalance, Proprioceptive integration, Motor cortex connectivity

## Abstract

**Background:**

Pisa Syndrome or Pleurothotonus is a relatively rare truncal dystonia, characterized by tonic flexion of the trunk and head to one side with slight rotation of the body. Since frequently associated to specific drugs such as antipsychotics and cholinesterase inhibitors or to Parkinson Disease, a pathophysiological role of cholinergic-dopaminergic imbalance has been suggested. We report here the first case of Pisa Syndrome due to an extracerebral pathology as subdural haematoma.

**Case presentation:**

A hypertensive patient was admitted to Our Department for subacute onset of tonic flexion and slight rotation of the trunk associated to progressive motor deficit in left upper limb after a mild head trauma without loss of consciousness occurred around three month before. No previous or current pharmacological interventions with antidepressant, neuroleptic or anticholinergic drugs were anamnestically retrieved. Familiar and personal history was negative for neurological disorders other than acute cerebrovascular diseases. Acutely performed cerebral MRI with DWI showed a voluminous right subdural haematoma with mild shift of median line. After surgical evacuation, both motor deficit and truncal dystonia were dramatically resolved. At one-year follow up, the patient did not develop any extrapyramidal and cognitive signs or symptoms.

**Conclusions:**

According to many Authors, the occurrence of truncal dystonia during several pharmacologic treatments and neurodegenerative disorders (such as Alzheimer disease and parkinsonian syndromes) supported the hypothesis that a complex dysregulation of multiple neurotransmitter systems are involved. We suggest a possible role of basal ganglia compression in pathogenesis of truncal dystonia by means of thalamo-cortical trait functional disruption and loss of proprioceptive integration. A further contribution of the subcortical structure displacement that alters motor cortex connectivity to basal ganglia may be postulated.

## Background

First described by Ekbom in early 1972 [1], Pisa Syndrome (PS) or Pleurothotonus is a relatively rare global dystonia characterized by tonic flexion of the trunk and head to one side with slight rotation of the body [2]. Female gender, old age and organic brain disorders such as parkinsonisms and dementia are considered its most common risk factors [2]. During past decades, it was report both in patients taking typical/atypical antipsychotic agents [3] and in idiopathic cases or during neurodegenerative diseases such as Parkinson Disease (PD) [4] and other parkinsonisms [5,6]. More recently, an association with cholinesterase inhibitors [7], selective serotonin re-uptake inhibitors and other antidepressants [8,9], antiepileptic as valproic acid [10] or antiparkinsonian agents as dopamine-agonists [11,12] has been accounted, suggesting a pathophysiological role of cholinergic-dopaminergic imbalance in the regulation of axial muscle tone [13]. To date, only one case of secondary PS unrelated to exposure to psychotropic medications has been reported in a patient with idiopathic normal pressure hydrocephalus (iNPH) in which a dysregulation of dopaminergic pathways has been observed [14]. We report here a case of PS in a patient with subacute subdural haematoma (SDH) that was reverted by evacuation intervention.

## Case presentation

**Figure 1:**
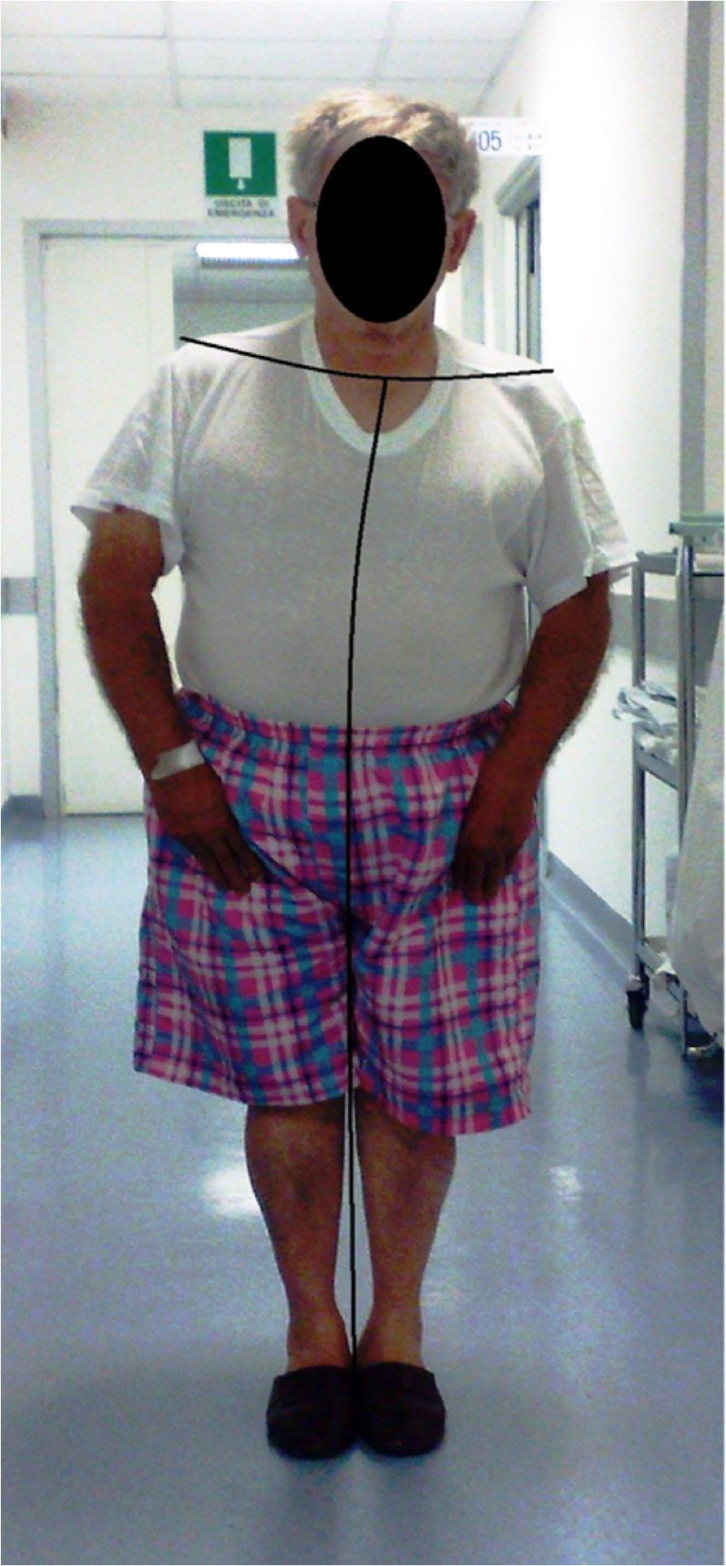
**Patient in standing position before intervention.cph.** A slight but sustained tonic flexion of the trunk to the left side was present in standing position with ipsilateral forward rotation. Vertical axis highlights the left lateral skew of the trunk. Horizontal line reflects the forward rotary motion of the left shoulder.

**Figure 2:**
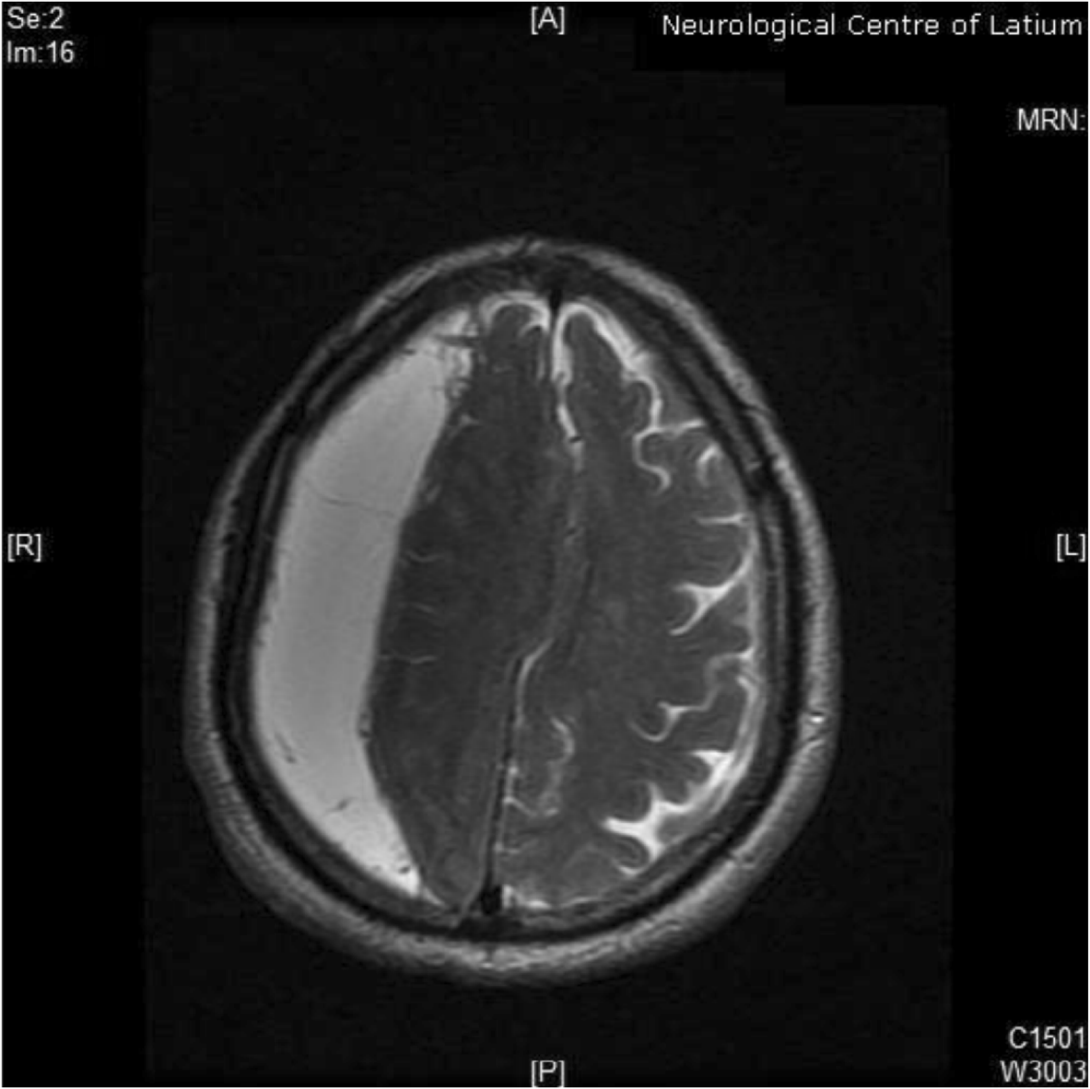
**Axial T2-weighted slice of cerebral MRI.** A voluminous right frontal, temporal and parietal subdural haematoma with a diameter of 28 mm was present with ipsilateral ventricle compression and an 8 mm shift of median line. A minimal amount of haematic shedding (3 mm diameter) was also present in left frontal subdural space. Some periventricular hyperintense areas were observed as probable expression of microvascular pathology.

**Figure 3:**
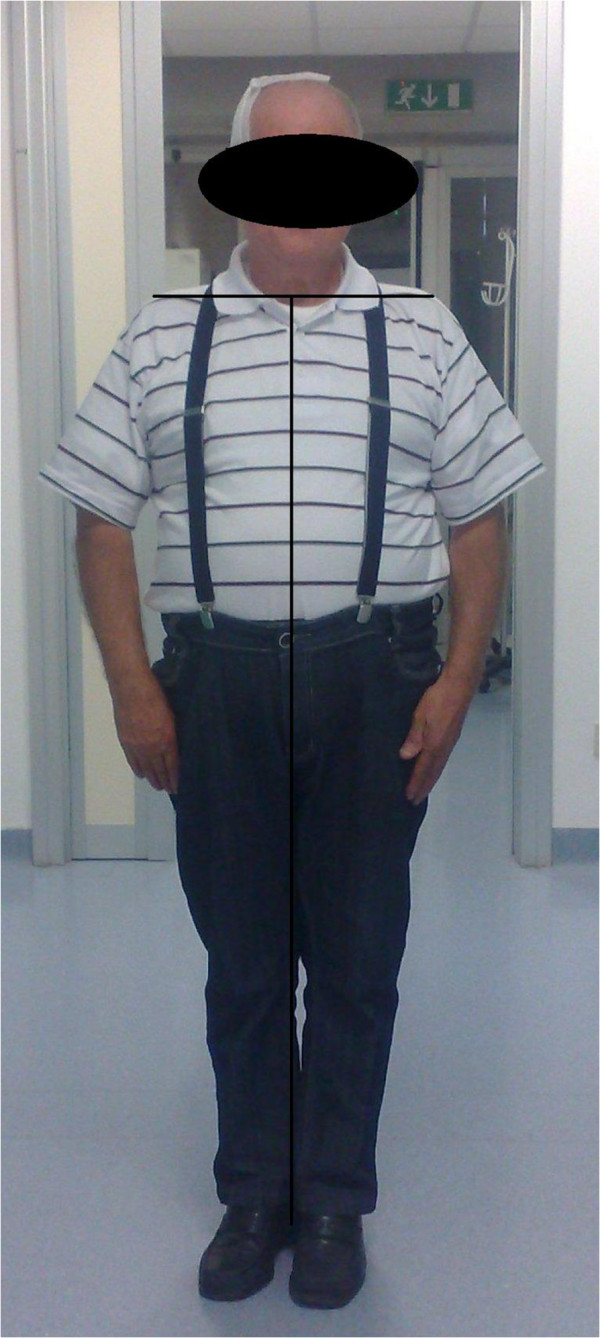
**Patient in standing position after intervention.** Four days after haematoma evacuation, both tonic trunk flexion and weakness in left upper limb dramatically improved until complete resolution. The patient maintained adequate standing position and did not swerve to the left during walking.

A 76 years-old hypertensive man was admitted to the Department of Neurosciences of Neurological Center of Latium in September for recent onset of slowly progressive weakness in left upper limb and postural instability. He also was observed walking with a tilt toward the left during the last week because of slight rotation of the trunk. The patient referred a mild head trauma without loss of consciousness occurred around three months before. No history of major operations, neurological disorders or drug and alcohol abuse was reported. Hypertension was treated by means of ramipril 5 mg/day and amlodipine 10 mg/day for more than 5 years with an optimal control of pressure values. No other previous or current pharmacological interventions were anamnestically retrieved, including with antidepressant, neuroleptic or cholinesterase inhibitors drugs. Family history was negative for neurological disorders other than acute cerebrovascular diseases. Both laboratory studies (liver and kidney function, serum electrolytes, lipids, coagulation, haemachrome) and electrocardiograms were within normal ranges. Neurological examination revealed motor deficits in left hand grip and forearm flexion associated to sustained tonic 15-degrees lateral flexion of the trunk to the left side with slight forward rotation (see Figure 1). Abnormal posture of the trunk worsened in walking and was reduced in lying. We also observed left lateropulsion and difficulty in maintain Romberg position after eyes closure. We did not find significant changes in muscle tone or limb agility impairment, such as cerebellar signs and symptoms were not elicited. Tremor was not present at rest or during action or postural maintaining. Acutely performed cerebral Magnetic Resonance Imaging (MRI) with Diffusion Weighted Imaging (DWI) showed a voluminous right frontal, temporal and parietal subdural haematoma with a diameter of 28 mm. Ipsilateral ventricle was compressed with 8 mm shift of median line (Figure 2). A minimal amount of haematic shedding (3 mm diameter) was also present in left frontal subdural space. In T2-weighted images, some periventricular hyperintense areas and a right cerebellar lacunar focus were observed as probable expression of microvascular pathology. After neurosurgical evaluation, the patient underwent surgical evacuation by means of single burr hole drainage under the local anesthesia and positioning of a soft silicon drain, which was removed within 2 days. Twenty-four hours after the intervention, neurological examination was negative and tonic flexion of the trunk was dramatically improved (Figure 3). We obtained postoperative Computerized Tomography scans within 3 days after surgery that showed a minimal residual hygroma. Four days after intervention, both dystonic deviation and lateropulsion disappeared and the patient was dismissed. No neurological deficit was evident at 12-months follow up, with particular regard of extrapyramidal and cognitive signs or symptoms.

## Conclusion

Although the pathophysiology of PS is poorly understood, the implication of monoamines has been widely suggested [13,15]. According to many Authors, the occurrence of truncal dystonia during several pharmacologic treatments and neurodegenerative disorders (such as Alzheimer disease and parkinsonian syndromes) supported the hypothesis that a complex dysregulation of multiple neurotransmitter systems are involved [13,16]. Both an excess of cholinergic transmission and a decrease of dopaminergic tone could be implied in some cases because of causative effect of cholinesterase inhibitors and/or antagonist effects of neuroleptics on type-2 dopamine (D2) and type-2 serotonin (5HydroxyTriptamine, 5HT-2) receptors [15]. Down-regulation of postsynaptic norepinephrine (NE) and 5HT-2 receptors induced by antidepressant drugs may also determine a dysfunction of brain stem pathways involved in axial tone control [17]. In parkinsonian syndromes, PS and other lateral truncal dystonia has been related not only to pharmacological treatment, but also to the clinical stage of the disease [18]. An imbalance in the dopaminergic-cholinergic system, depending both on the progression of nigrostriatal pathology and on pharmacological treatment has been postulated in PD [18,19]. Supporting the neurotransmitters hypothesis, efficacy of anticholinergic drugs and switching therapy to less cholinergic antipsychotics, such as quetiapine or clozapine has been described in some reports [15]. To our knowledgement, this is the first case of secondary PS not related to both pharmacological intervention and neurodegenerative disorders. In our patient, there was no personal or family history of cerebral degenerative disorders or specific previous and current pharmacological treatments other than antihypertensive drugs ramipril and amlodipine. Otherwise MRI revealed wide compression phenomena of subcortical structures and a significative shift of median line. The previously reported case of PS with no previous exposure to specific drugs was related to an iNPH in which brain asymmetry seems to affect neurotransmitters function by means of a downregulation of D2 receptors in the striatum due to nigrostriatal dopaminergic pathways alteration [14,20,21]. In this case, a possible role of functional disruption of non-dopaminergic systems such as thalamo-cortical tract through the corona radiata may be hypotized. Experimental results suggest that axial posture impairment in PD are related to progressive loss of propioceptive integration during spatial orientation tasks despite the integrity of vestibular system [22]. Some Authors suggested that an imbalance between control mechanisms of postural orientation and stabilization might explain both dystonic phenomena and instability in PD patients [4,19,22]. In our case, tonic flexion of the trunk was indeed associated to left lateropulsion and static and dynamic postural instability suggesting such an impairment of sense of position and more generally a lateralized propioceptive dysfunction [22]. Compressive effect of SDH on the afferent fibers to primary somatosensory cortex from ventral posterolateral nucleus of the thalamus may account for a subtle loss of the sense of position and movement and an impairment of proprioceptive integration resulting in an impairment of the control of body orientation [16,21]. Furthermore, a complex contribution of basal ganglia in postural control and body rotation may be postulated according to several lesion studies that accounted for uncontrolled axial posture after controlateral lesion of caudate and lenticular nucleus [4,18]. Displacement of subcortical structure in our patients may alter motor cortex connectivity to basal ganglia with consequent reduction of inhibition pathways to sensory-motor cortex because a particular involvement of unilateral caudate and lenticular nuclei [4,16,22].

## Consent

Written informed consent was obtained from the patient for publication of this Case report and any accompanying images. A copy of the written consent is available for review by the Editor of this journal.

## Abbreviations

PS: Pisa Syndrome; PD: Parkinson Disease; iNPH: idiopathic Normal Pressure Hydrocephalus; SDH: Subdural haematoma; MRI: Magnetic Resonance Imaging; DWI: Diffusion Weighted Imaging; D2: type-2 dopamine receptors; 5HT: type-2 5-HydroxyTriptamine; NE: norepinephrine receptors.

## Competing interests

The authors declare that they have no financial and non-financial competing interests.

## Authors’ contributions

PM performed the preoperative and postoperative clinical evaluation of the patients, collected both literature and patient data and imaging and drafted the manuscript; AS interpreted neuroimaging, carried out neurosurgical intervention as first operator and revised critically the manuscript; MV performed the clinical follow up at one year and helped to collected literature data and to draft the manuscript; Cristiano Giannone carried out the neurosurgical intervention as second operator and helped to draft the manuscript; FDA collected anamnestic data, helped the interpretation of neuroimaging and participated in the manuscript draft; GM interpreted neuroimaging, performed preoperative and postoperative clinical evaluation, revised critically the manuscript and finally approved the version to be published. All authors read and approved the final manuscript.

## Pre-publication history

The pre-publication history for this paper can be accessed here:

http://www.biomedcentral.com/1471-2377/14/149/prepub
